# Development and Application of a Functional Human Esophageal Mucosa Explant Platform to Eosinophilic Esophagitis

**DOI:** 10.1038/s41598-019-41147-8

**Published:** 2019-04-17

**Authors:** Richard C. Kurten, Renee Rawson, Tetsuo Shoda, Loan D. Duong, Dolapo Adejumobi, Rebecca Levy, Robert O. Newbury, Marc E. Rothenberg, Praveen Akuthota, Benjamin L. Wright, Ranjan Dohil, Stacie M. Jones, Seema S. Aceves

**Affiliations:** 1 Department of Physiology & Biophysics, University of Arkansas for Medical Sciences, Little Rock, Arkansas USA; 2 Division of Allergy & Immunology, University of Arkansas for Medical Sciences, Little Rock, Arkansas USA; 3 Arkansas Children’s Research Institute, Little Rock, Arkansas USA; 4 Division of Allergy, Immunology, Department of Pediatrics, University of California San Diego and Rady Children’s Hospital, San Diego, California, USA; 5 Division of Allergy, Immunology, Cincinnati Children’s Hospital Medical Center, Cincinnati, Ohio USA; 6 Department of Pathology, University of Arkansas for Medical Sciences, Little Rock, Arkansas USA; 7 Department of Pathology, University of California, San Diego and Rady Children’s Hospital, San Diego, California USA; 8 Division of Pulmonary, Critical Care, and Sleep Medicine, Department of Medicine University of California, San Diego, California USA; 9 Division of Allergy, Asthma and Clinical Immunology, Department of Medicine, Mayo Clinic Arizona, Scottsdate, Arizona, Division of Pulmonology, Phoenix Children’s Hospital, Phoenix, Arizona USA; 10 Division of Gastroenterology University of California San Diego, San Diego, California USA; 11 Department of Medicine University of California, San Diego, California USA

**Keywords:** Gastrointestinal models, Pathogenesis

## Abstract

There is an increasing prevalence of esophageal diseases but intact human tissue platforms to study esophageal function, disease mechanisms, and the interactions between cell types *in situ* are lacking. To address this, we utilized full thickness human donor esophagi to create and validate the *ex vivo* function of mucosa and smooth muscle (n = 25). Explanted tissue was tested for contractile responses to carbachol and histamine. We then treated *ex vivo* human esophageal mucosa with a cytokine cocktail to closely mimic the Th2 and inflammatory milieu of eosinophilic esophagitis (EoE) and assessed alterations in smooth muscle and extracellular matrix function and stiffening. We found that full thickness human esophagus as well as the individual layers of circular and longitudinal muscularis propria developed tension in response to carbachol *ex vivo* and that mucosa demonstrated squamous cell differentiation. Treatment of mucosa with Th2 and fibrotic cytokines recapitulated the majority of the clinical Eosinophilic Esophagitis Diagnostic Profile (EDP) on fluidic transcriptional microarray. Transforming growth factor-beta-1 (TGFβ1) increased gene expression of fibronectin, smooth muscle actin, and phospholamban (p < 0.001). The EoE cocktail also increased stiffness and decreased mucosal compliance, akin to the functional alterations in EoE (p = 0.001). This work establishes a new, transcriptionally intact and physiologically functional human platform to model esophageal tissue responses in EoE.

## Introduction

Food transit through the esophagus involves a complex interplay of mucosa to provide lubrication and barrier function, muscle layers for propulsion, and enteric and central innervation for coordinated transport of food to the stomach. Proper function is essential to adequate nutritional intake. A rise in esophageal disorders including gastroesophageal reflux, EoE^1^, and esophageal carcinoma^2^ drives a need for human disease-relevant experimental platforms.

While murine models can recapitulate human disease, in the esophagus there are a number of structural cell differences between species^3^. The human esophagus transitions to smooth muscle more proximally than the murine esophagus. The epithelium is keratinized in mice but not in humans. The innervation of the human and murine esophagus is distinct so motility disturbances can be challenging to assess in mice^3^. These structural and functional differences combined with evolving questions of general translatability of murine disease models to human polygenic diseases underscores a need for intact human platforms to experimentally manipulate human esophageal cells within their natural environment. Feline, opossum and guinea pig esophageal models have been utilized to study esophageal function^4,5^, and these species are closer to the human esophagus in terms of structural cells and innervation, yet they remain distinct species and the study of human esophageal disease would generally benefit from a widely applicable human esophageal platform.

One esophageal disease of rising prevalence is EoE. EoE is diagnosed when ≥15 eosinophils per high power field are present in the esophageal epithelium^1,6^. Progressive tissue remodeling with fibrosis in children and adults causes esophageal rigidity, lost tissue compliance, and resultant food impactions and strictures^7–13^. Dysmotility is an increasingly appreciated consequence of unbridled Th2 inflammation in EoE^11,14–19^. Current investigational platforms for EoE include small biopsies^13,19^, isolated structural cells^13,19,20^, spheroids^21,22^, and immortalized epithelial cells^23,24^. While studies of spheroids are feasible and represent 3-dimensional tissues, they require dispersion and re-juxtapositioning of cells and therefore cannot entirely represent the function of an intact epithelium or its interactions with neighboring cells such as smooth muscle^21,23,25^.

To address the experimental need for a human esophageal platform, we systematically characterized the baseline and induced functional and transcriptional alterations in intact human tissue cultured *ex vivo*. We utilized full thickness mucosal tissue and isolated longitudinal and circular smooth muscle bundles in functional tension response studies. Treatment of intact mucosa with a cytokine cocktail containing Th2 and pro-fibrotic cytokines increased mucosal stiffness and induced an EoE-like gene transcription profile. To our knowledge, this is the first utilization of intact human esophageal tissue explants to model a disease state both physiologically and transcriptionally.

## Results

### Isometric tension studies

#### Intact esophageal rings

**Figure 1:**
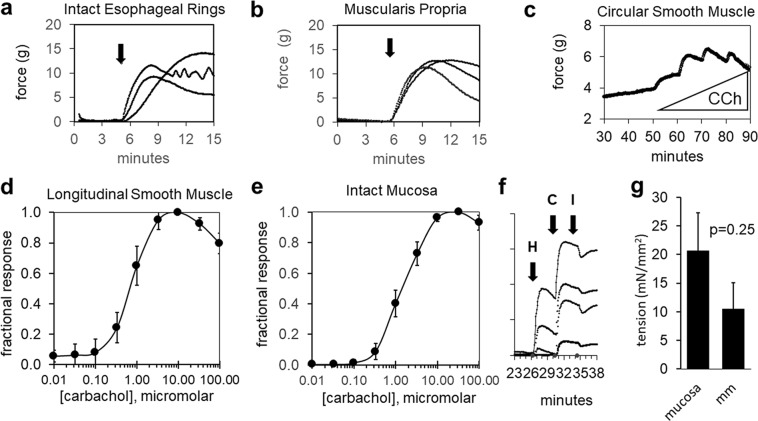
Induced force responses in human esophageal smooth muscle. Contractile forces were generated in response to carbachol under isometric conditions in (**a**) freshly isolated intact tissue rings (n = 3 donors) and (**b**) muscularis proria rings after storage at 7 °C for 24 hours (4th donor). Arrows indicate carbachol addition. Carbachol induces concentration-dependent force responses in: (**c**) isolated circular smooth muscle rings, (**d**) longitudinal smooth muscle bundles (n = 4, cultured for 1 day), and (**e**) mucosal strips (n = 4, cultured for 1 day). (**f**) Histamine (H) stimulates LSM bundle contraction independently of carbachol (C) and forces are attenuated by isoproterenol (I) (4 bundles, cultured for 3 days). (**g**) Induced tension responses (1 μM carbachol) in microdissected MM (p = 0.25, n = 8, cultured 3–5 days).

Carbachol treatment of intact rings from the distal half of the human esophagus hung immediately after preparation increased isometric force (Fig. 1a, arrow). This response was retained after storage at 7 °C for 24 hours and persisted after removal of the mucosal layer (Fig. 1b). Responses were characterized by a peak within minutes that decayed to a stable plateau. These results indicate esophageal tissues retain contractile function *ex vivo* for at least 24 hours post acquisition.

#### Individual tissue layers

The inner circular smooth muscle (CSM) layer of the muscularis propria is oriented in the appropriate direction for tension development in esophageal rings. To confirm that CSM generated the majority of the observed tension in the full thickness esophagus, we dissected away the mucosa and found that contractile response to carbachol was retained (Fig. 1c).

Diffusion limited nutrient and metabolite exchange precluded long-term *ex vivo* culture of intact esophageal rings. Moreover, estimates of the tension contributions by longitudinal smooth muscle (LSM) and muscularis mucosa (MM) (where the muscle is longitudinally oriented) cannot be determined in rings due to tissue orientation. We therefore isolated tissue strips (~15 × 1 × 1 mm, Fig. S2) from each esophageal layer (mucosa, LSM, and CSM), cultured them for 1–5 days as indicated, and transferred them to organ baths for measurement of isometric tension responses. Carbachol stimulated dose-dependent increases in isometric force in LSM stretched longitudinally (Fig. 1d). Mucosa also generated tension along the longitudinal axis upon treatment with carbachol (Fig. 1e), consistent with the orientation of the MM. Histamine induced LSM tension that was augmented by 10 μM carbachol and reduced by the β-agonist isoproterenol (Fig. 1f). Carbachol also induced isometric tension in microdissected MM preparations (Fig. 1g). By contrast, in mucosa stretched along the circular axis, carbachol did not induce isometric tension (data not shown). These results indicate that the three smooth muscle layers of the human esophagus as well as the full thickness mucosa can be separated and maintained to retain their contractile responses *ex vivo* for at least 3–4 days.

### Analysis of tissue stiffness

**Figure 2:**
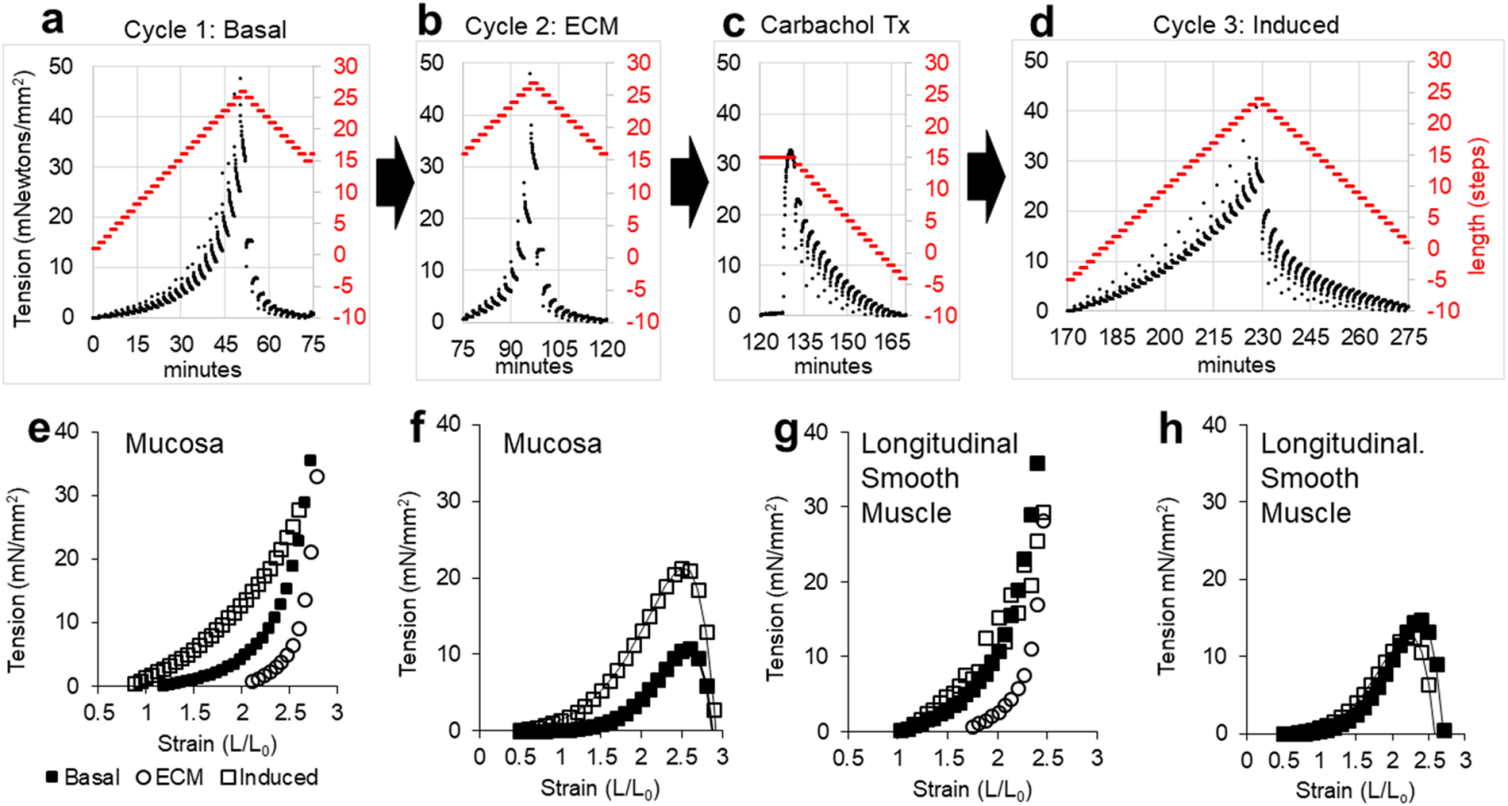
Tension versus strain measurements to determine stiffness. After culture for 3 days, tension measurements were collected from intact mucosa during sequential cycles (**a,b**) of stepwise lengthening followed by stepwise shortening of the same esophageal mucosa strip. Lengthening was reversed at a force threshold set to avoid tissue damage. Shortening was reversed as the force approached zero. The step tracings indicate the length (L, right y-axis) at which the tissue was held for 2 minute intervals as length was changed in 0.72 mm increments. (**a**) In strain cycle 1, tissue tension (black line) but not length (red line) reached baseline on stepping down and (**b**) this remained the case in strain cycle 2. (**c**) 1 μM carbachol increases tension and shortening to less than the starting length. (**d**) In strain cycle 3 the tissue shortens to the starting length. (**e**) Integrated tension measurements plotted as a function of strain. (**f**) Plots generated from panel e data by subtracting cycle 2 from cycle 1 (basal) or cycle 3 (induced). (**g**) LSM bundle tension versus strain plots collected after culture for 10 days. (**h**) Contribution of smooth muscle to tissue tension in in basal and induced states for the LSM in panel g.

Some esophageal disorders such as EoE increase esophageal rigidity. To understand if we could induce functional alterations in the *ex vivo* platform that partially mimic a chronic esophageal disease, we assessed esophageal stiffness in the intact mucosa after culture for 3 days. We began by characterizing the baseline stiffness of individual tissue layers using sequential cycles of lengthening. In cycle 1 (Fig. 2a), the lengthening of mucosal tissue was only partly reversible since the tissue failed to return to its starting baseline length (red line). In cycle 2 (Fig. 2b), lengthening was reversible although the starting length was about twice that for cycle 1. When treated with 1 μM carbachol after cycle 2 (Fig. 2c), the mucosal tissue generated force and shortened to less than the starting length for cycle 1 at preload tension. In tension versus strain measurement cycle 3 (Fig. 2d), the lengthening was completely reversible.

The data in Fig. 2a–d were reduced to time-integrated tension versus strain curves for mucosal lengthening (Fig. 2e) with tissue stiffness indicated by the slope of the curves. The curves were never linear, indicating that stiffness is length-dependent and that mucosal tissue is a hyperelastic material like rubber on lengthening. However, by contrast to rubber, the stretched mucosa failed to return to its original length on shortening in cycle 1 (Fig. 2b), leading to a distinct rightward shift in the tension versus strain curve for lengthening in cycle 2 (Fig. 2e). While tissue in the basal state (cycle 1, black squares) exhibits increasing stiffness gradually as it is stretched from L_0_ to 2.5x L_0_, the same tissue in cycle 2 (open circles) is infinitely compliant (stiffness = 0) from L_0_ to 2x L_0_, and rapidly stiffens from 2x to 2.5x L_0_. These observations led us to hypothesize that two additive elements contribute to the stiffness of the human esophageal mucosa. One element, lost by stretching the tissue, is contributed by smooth muscle. Another element, revealed in cycle 2 is likely a cross-linked biological polymer of the mucosal extracellular matrix, such as collagen-1. If basal stiffness is a combination of smooth muscle and extracellular matrix, then carbachol treatment to increase muscle force generation should restore the stiffness lost in the region of 1x to 2x L_0_ after stretch cycle 1. Indeed, treatment with carbachol resulted in a convincing leftward shift of the tension versus strain curve for cycle 3 (open squares, Fig. 2e). As further confirmation for the proposed role of smooth muscle in restoring of basal tissue stiffness after stretching, we subtracted the cycle 2 (extracellular matrix) length versus tension curve from the cycle 1 (basal) curve or from the cycle 3 (induced) curve (Fig. 2f). The difference curves exhibited peaks of maximum tension development at ~2.5x L_0_. Such curves are characteristic of active force generation by muscle cells^26,27^ where maximal tension corresponds to maximal overlap of force generating myosin and actin filaments. LSM bundles exhibited similar properties after culture for 10 days (Fig. 2g,h) with maximum basal and induced tension more similar than observed for mucosa.

**Figure 3:**
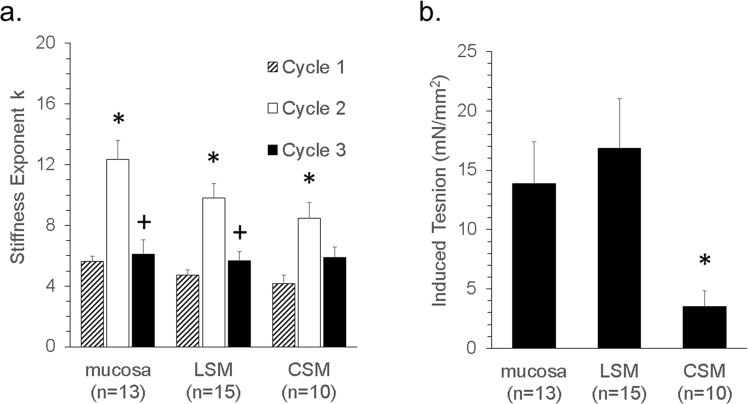
Stiffness and induced tension in *ex vivo* esophagus tissues. (**a**) Stiffness exponent k for strain cycle curves fit by tension = A(L/L_0_)^k^ for basal (cycle 1), extracellular matrix (cycle 2), and carbachol induced (cycle 3) tissue stiffness (*p < 0.05 vs Cycle 1, ^+^p < 0.05 vs. Cycle 2.) and (**b**) the corresponding 1 μM carbachol-induced tension response (*p < 0.05 vs mucosa and lsmb). The data are pooled from samples cultured for 1–14 days (mean 5.3 days for mucosa, 5.2 days for LSM and 4.6 days for CSM) before measuring stiffness. where n represents the number of donors used for each tissue. *p < 0.05 vs Cycle 1, ^+^p < 0.05 vs. Cycle 2.

The tension versus strain data was best fit by a power function: Tension = A (L/L_0_)^k^ where A is the resting tension, L/L_0_ is the strain, and k > 1 indicates tissue stiffening on lengthening. Tissue stiffness is readily calculated from the derivative of the power function (=Ak(L/L_0_)^k−1^) where Ak gives the stiffness at L_0_. While both A and k contribute to measured stiffness, the calculated value for A is dependent on an accurate measurement of L_0_ whereas the value calculated for k is independent of L_0_. Accurately measuring L_0_ is a challenge, so k is the most reliable parameter for statistical comparisons. We observed significant increases in k after stretching (Fig. 3a, clear bars) that could be restored to near basal values by treatment with contractile agonist in each esophageal layer (Fig. 3a, black bars). The magnitude of tension responses to carbachol were variable, with significantly less carbachol induced tension in circular smooth muscle than in longitudinal smooth muscle or in mucosa (Fig. 3b).

Taken together, A and k in the power function accurately model the composite mechanical stiffness properties of smooth muscle and extracellular matrix. In the absence of smooth muscle contractile system activation, tissue stretches with little resistance to strain (A is small, tissue is compliant) until it reaches a threshold between 1.5 and 2.5 times resting length where it becomes very stiff as the limits to ECM strain are reached. In the presence of basal or induced smooth muscle tone, the tissue becomes stiffer at the resting length, and hence becomes less distensible. Together these results reveal that 1) combined properties of extracellular matrix and smooth muscle determine the composite stiffness of the esophagus, 2) acetylcholine and histamine acutely regulate stiffness through the actin-myosin machinery, and 3) we can use this approach to characterize both extracellular matrix and smooth muscle contributions to human esophageal muscle tissue tension and stiffness.

### Inducing a disease phenotype *ex vivo*

**Figure 4:**
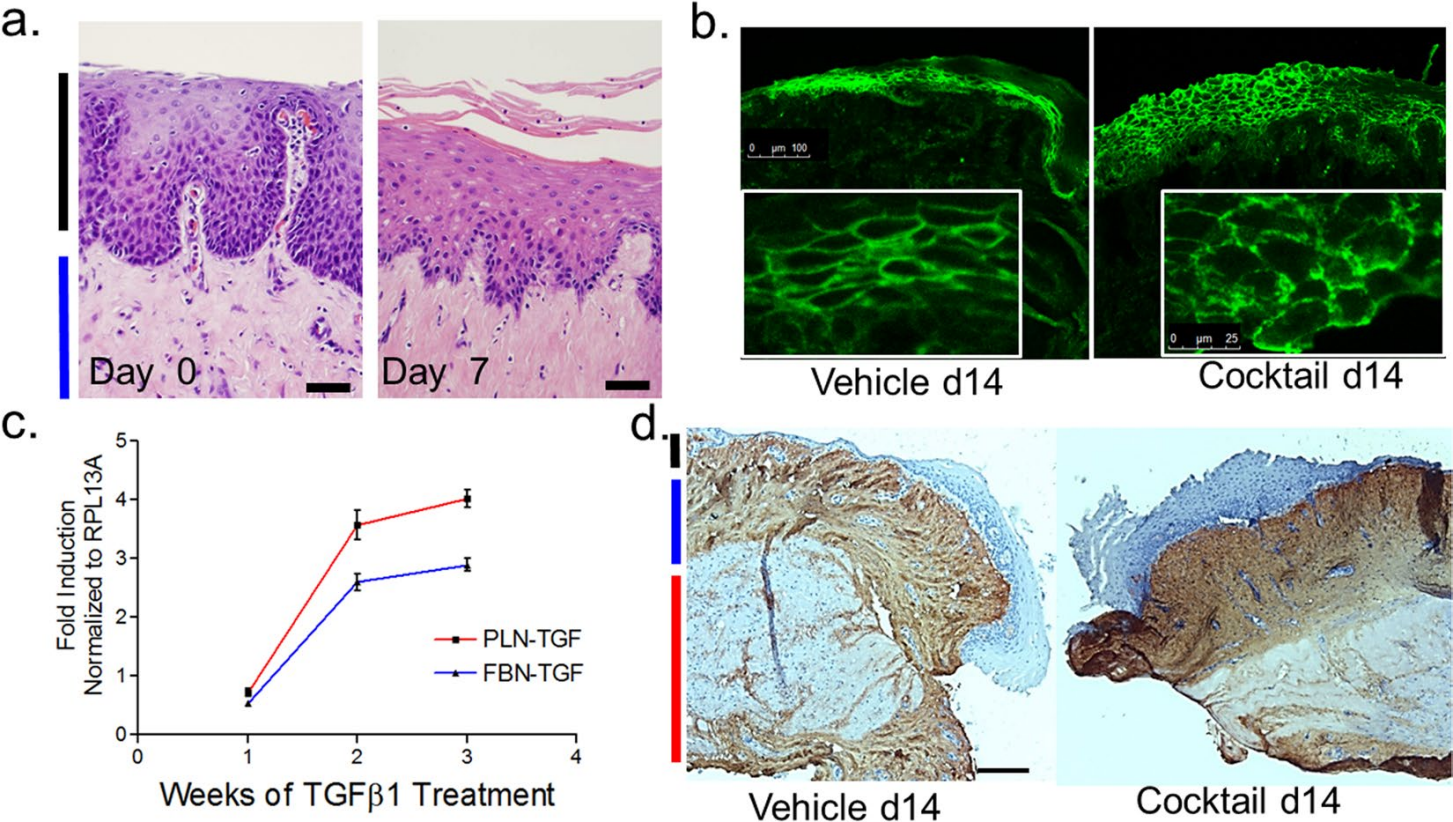
Properties of esophagus *ex vivo*. (**a**) Regeneration of superficial squamous cells of the stratum spinosum on day 7 after gentle scraping on day 0 (both untreated). (**b**) E-cadherin staining demonstrates squamous epithelial differentiation in full thickness esophageal mucosal explants cultured *ex vivo* for 14 days in vehicle or Th2 cytokine cocktail. 14 days of Th2 cytokine cocktail causes disruption of the epithelial barrier as occurs in the *in vivo* EoE active disease state. (**c**) Gene expression of phospholamban (PLN) and fibronectin (FBN) in LSM over 4 weeks of TGFβ1 treatment (***p < 0.0001). (**d**) Collagen1 staining in human esophageal mucosa following 14 days of vehicle or Th2 cocktail treatment (black line: epithelium, blue line lamina propia). Scale bars represent 50 (**a**) and 100 (**d**) microns if not otherwise labeled.

In addition to retention of smooth muscle function, the basal epithelial cell layer of mucosal explants was also preserved when assessed histologically. Sloughed squamous cells were evident on the explants and accumulated in the bottom of the culture dish. To determine if this accumulation represented ongoing squamous differentiation, we gently scraped the surface on an explant to remove the superficial squamous epithelial cells on day 0 and found that they reaccumulated over 7 days in culture (Fig. 4a).

To validate the potential disease relevance of the *ex vivo* esophageal platform, we utilized a Th2 cytokine cocktail that closely mimics an EoE-like inflammatory milieu to treat mucosal explants. Immunofluorescence staining for E-cadherin (Fig. 4b) further confirmed sustained squamous differentiation after 14 days with junctional protein reorganization induced by the cytokine cocktail^28^. TGFβ1 treatment of LSM bundles also significantly induced fibronectin and phospholamban expression from 1 to 3 weeks in culture (p < 0.001) (Fig. 4c).Histologically, collagen I was induced in full thickness *ex vivo* mucosa after 14 days of treatment with the EoE-like cytokine cocktail as compared with vehicle (Fig. 4d).

**Figure 5:**
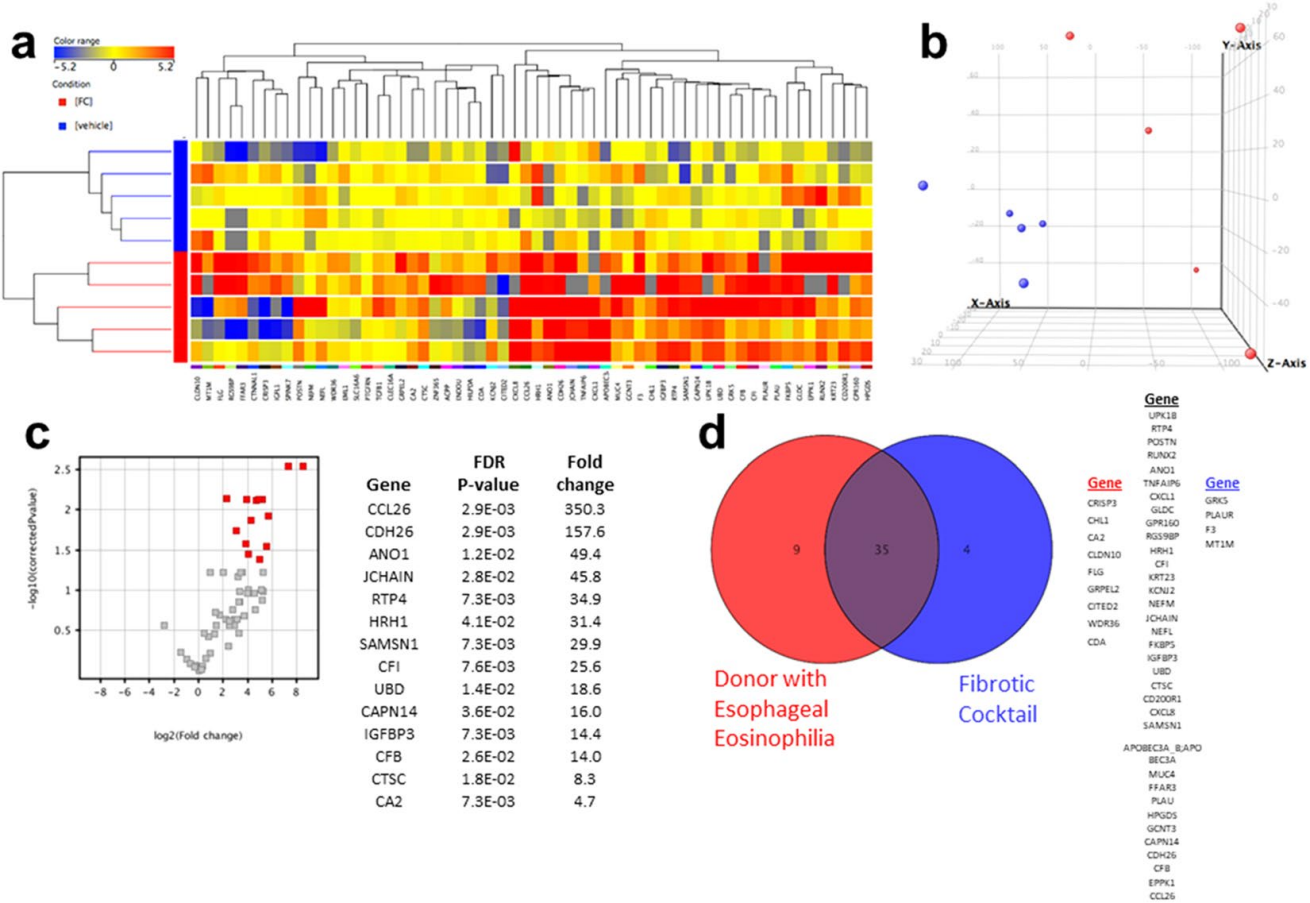
Comparison of the esophageal transcriptome in vehicle treated (blue) and Th2 cytokine cocktail (red). Color range is based on log2 normalized intensity (**a**). Three-dimensional plot containing five paired samples (red = cocktail, blue = vehicle) derived from principal component analysis of the genes shown in the heat map in order assess the geometric distance between the samples (**b**). Volcano plot of genes in the EDP with FDR p < 0.05, fold change >2 (red, left panel) following treatment with Th2 cytokine cocktail (**c**). Venn diagram comparing the number of genes identified as dysregulated (>5 fold change) in both an organ donor esophagus with eosinophilia and samples treated with the cytokine cocktail. Unique and overlapping genes are listed in the right panel (**d**). Mucosa was treated for 72 hours (one paired sample) to 7 days (4 paired samples).

To demonstrate that the cytokine cocktail induced a molecular EoE-like disease state *ex vivo*, we utilized the Eosinophilic Esophagitis Diagnostic Panel (EDP), a validated transcriptional profile of 96 genes that are dysregulated in EoE. Due to the archiving time for some samples, a 50% call rate filter was applied to the 96 genes to focus on the informative genes, resulting in a cluster of 60 genes. These data demonstrated that the vehicle treated mucosa retained transcription of esophageal genes while the Th2 cytokine cocktail treated mucosa induced a pattern of gene expression that aligned with the *in vivo* EoE state (Fig. 5a)^29^. Principal component analysis demonstrated separation of gene expression between the vehicle and cocktail treated mucosa and significant dysregulation of a number of EoE-specific genes (Fig. 5b,c). Using a donor whose esophagus met histologic criteria for EoE (epithelial eosinophils of 45 per high power field, positive basal zone hyperplasia, 23 eosinophils per high power field in the lamina propria, fibrosis score of 2), we compared the genes expressed in this donor with the 10 paired vehicle and cytokine-treated mucosa. This analysis of genes induced >5-fold demonstrated that the inherent esophageal eosinophilic state (red) closely aligned with the EoE stated induced *ex vivo* (blue) by the EoE-cocktail (Fig. 5d).

To better replicate an EoE-like tissue microenvironment, we modelled adoptive transfer techniques used in mice^30,31^ by injecting isolated human peripheral blood eosinophils into the bottom surface of mucosal explants. After injection, an eotaxin-1 soaked sponge was laid on the epithelial surface to promote chemotaxis of the injected eosinophils. Following 24–72 hours of incubation, eosinophil infiltration, as verified using an eosinophil peroxidase (EPX) stain, was present in the lamina propria and epithelium (Supplemental Fig. 1). While we have not evaluated secretory properties of the infiltrating eosinophils, these data demonstrate the feasibility of creating structural cell dysregulation and an inflammatory environment *ex vivo* that is similar to EoE.

**Figure 6:**
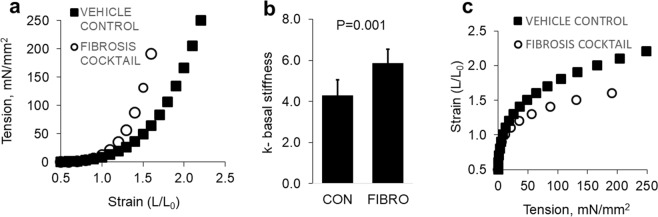
Stiffening esophageal mucosa *ex vivo* with the Th2 cocktail. (**a**) Th2 cytokine cocktail shifts the tension versus strain to the left indicating shortening and mucosal stiffness. (**b**) Basal stiffness exponent k is significantly increased in mucosal strips treated with Th2 cytokine cocktail. (**c**) Treatment with Th2 cytokine cocktail reduces mucosal compliance.

Functionally, culture of mucosal strips with the Th2 cytokine cocktail induced a significant leftward shift in the basal tension versus strain curve after 12–15 days (Fig. 6a). Cocktail-treated as compared to vehicle treated strips had a significant increase in tissue stiffness at L = 1.25 L_0_ as deduced from the value for k (p < 0.001) (Fig. 6b). If the averaged stiffness plot is inverted to convey the compliance of the tissue studies (Fig. 6c), the data resembles the compliance curves derived from *in vivo* EoE endoscopic functional lumen imaging probe (endoFLIP)^32^. Taken together, these data show that treatment of LSM bundles and full thickness esophageal mucosal strips *ex vivo* with a cytokine cocktail similar to the inflammatory cascade seen in EoE can induce alterations in structural cell gene transcription and function that aligns with the disease state.

## Discussion

In this manuscript, we describe a novel experimental platform to study both baseline and cytokine-modified function of human esophageal mucosa and smooth muscle and apply it to recapitulate an EoE-like disease state. We reasoned that the mucosa was the most relevant tissue for modelling aspects of EoE as it is the tissue studied in patient biopsies. Unlike cell culture or organoid culture, the use of intact mucosal tissue obviates the need for cellular disruption and is more likely to accurately translate to the *in vivo* cell-cell interaction state. We also investigated the mechanical properties of the human esophageal mucosa and muscularis mucosa which have not been previously measured *ex vivo*. Many studies have focused on lower esophageal sphincter function^33,34^, and a single study reports that human esophageal CSM and LSM are remarkably compliant with maximal tension development at twice the resting length^35^. We confirmed these findings and further demonstrated that the esophageal mucosa was highly compliant at baseline and that both isolated smooth muscle bundles and intact mucosa responded functionally to the acetylcholine analogue carbachol even after culture for many days.

The value of this approach is supported by the ability of our esophageal mucosa platform to mimic EoE at the levels of global transcriptional induction as assessed by the EDP transcription profile and alterations in esophageal stiffness. Treatment with an EoE-like Th2 cytokine cocktail induced disease-relevant gene transcription, alterations in E-cadherin organization of the epithelial barrier as occurs *in vivo* in the active EoE disease state^28^, and also increased mucosal stiffness leading to compliance properties similar to those obtained from EoE patients during endoFLIP^36^. Given this applicability of the platform to one esophageal disease, we suggest that our studies could help investigators to elucidate esophageal function in response to various stimuli including cytokines, acid, carcinogens, and therapeutic drugs. Since the mucosa and muscle bundles are in an intact tissue, cell-cell and cell-matrix communication is not breached which may lend a deeper understanding of tissue responses. Indeed, our studies indicate that changes in mucosal stiffness can be mediated, at least in part, by alterations in smooth muscle contraction. Although a simplification of the underlying processes, our results point to 2 distinct additive mechanisms with the potential to account for increased stiffness in the EoE esophagus – (1) altered smooth muscle stiffness due to altered actin-myosin (revealed by responses to carbachol and histamine) and (2) altered extracellular matrix stiffness (revealed by loss of smooth muscle-mediated elastic recoil with strain).

By using exogenous recombinant Th2 and fibrotic cytokines, we have tried to closely recapitulate the cytokine milieu of EoE as proof of principle that the *ex vivo* human mucosa could be used to study disease specific pathways. However, we acknowledge the weaknesses in our current system including the lack of infiltrating inflammatory cells, a failure to maintain/reconstitute a thick squamous layer, and the lack of altered extracellular matrix thickness visible on hematoxylin and eosin staining. These issues could be addressed in the future by using longer term culture, by injecting and promoting transmigration of inflammatory cells into the tissue (which we have shown herein is feasible for eosinophils), and by the activation of mast cells *in situ* using anti-IgE antibodies.

Our *ex vivo* culture approach is also limited to the analysis of individual tissue layers (mucosa, CSM, LSM) to permit adequate gas, nutrient and metabolite exchange between the tissue and medium. An implicit assumption in our approach is that studies using human tissue will yield results more translatable to human disease than studies in non-human model organisms like the mouse. While demonstration of this being the case or not is beyond the scope of this manuscript, we can say that a human EDP is not directly applicable to mice. Conversely, targeted gene disruption in human tissue explants is not efficient. Thus, the strongest experimental approaches will likely couple studies in mice and humans.

Our experiments demonstrate the feasibility of the human tissue approach for EoE. Although human tissue acquisition is unpredictable, the ability to maintain functional responses to carbachol and histamine over days to weeks allow experimental flexibility in timing. In addition, tissues remained functional despite transport from Arkansas to San Diego, allowing collaboration between labs at large distances. Future experiments to further validate the platform are warranted. We think our ability to experimentally model an EoE-like disease state *ex vivo*, using shared tissue should promote further collaborative efforts between esophageal disease investigators.

## Methods

### Human esophagus acquisition

Viable human esophagi were procured by the National Disease Research Interchange or the Arkansas Regional Organ Recovery Agency from deceased organ transplant donors. Criteria for organ selection included <10 pack year history of smoking, no cancer, no infection and no more than 2 chronic diseases reported. Esophagi were packaged and transported under transplant conditions to the Kurten laboratory within 24 hours. The acquisition and use of tissue from cadaveric donors was not found to constitute human subjects research (University of Arkansas for Medical Sciences IRB) and was IRB exempt (University of California, San Diego). The donor demographics are summarized in Supplemental Table 1. Individual donor characteristics and their use in experiments is detailed in supplemental Table 2.

#### Human esophageal tissue preparations

The distal two-thirds of the human esophagus were excised in the Kurten laboratory. The esophagus initially was cut into 5 cm thick rings. These rings were used intact or separated into longitudinal (LSM) and circular smooth muscle (CSM) rings for isometric tension measurements.

#### Mucosal Strip and Muscle Bundle Preparations

The distal 6–9 cm of the esophageal body 1 cm above the gastroesophageal junction and below the skeletal muscle (recognizable as red longitudinal bundles) was excised, cut longitudinally, placed mucosal side up, washed extensively, and cut laterally into ~15 mm wide large strips of mucosa with associated muscularis propria (Fig. S2). The intact mucosal layer (epithelium, subepithelial lamina propria, and muscularis mucosa [MM]) was separated from the muscularis propria by sharp dissection through the Meissner’s plexus. These mucosal strips (15 × 60 × 4 mm) were cut into smaller strips (1–2 mm wide) along the longitudinal axis of the esophagus after removing residual Meissner’s plexus.

Adventitia was dissected away from the muscularis propria under a dissecting microscope to reveal the underlying LSM bundles which were separated from the CSM via dissection through the myenteric (Auerbach’s) plexus. Dissected tissues were either cultured immediately or stored for 1–4 days at 7 °C in an organ preservation solution (SPS-1, Organ Recovery Systems, Chicago, Il)^37^.

### *Ex vivo* culture

Full thickness mucosa, isolated MM, LSM, and CSM were cultured in 24-well plates in 300  μL/well DMEM:F12 changed every 2–3 days and supplemented with penicillin (100 units/ml), streptomycin (100 µg/ml), Amphotericin B (0.25 µg/ml), and 0.5% Primocin (InvivoGen, San Diego, CA) at 37 °C, 5% CO_2_ in a humidified incubator with continuous agitation. The Th2-fibrosis cocktail contained: TGFβ1 (1 ng/ml), IL-13 (5 ng/ml), IL-4 (1 ng/ml), IL-2 (10 ng/ml), IL-9 (10 ng/ml), LIGHT/TNSF14 (10 ng/ml), histamine (1 μM) in DMEM:F12 supplemented with bovine serum albumin (1 mg/ml), which also served as the vehicle control.

### Measurements of tension and tissue stiffness

#### Tension measurements

Muscle bundles or mucosal strips were hung vertically from Radnoti (Monrovia, CA) force transducers (range 0–20 g) with a stiff wire in 35 ml tissue baths (Krebs Henseleit buffer, Sigma Chemical Company, St Louis, Mo, aerated at 37 °C) for isometric force measurements along the long axis of the tissue. The force transducer output was zeroed and samples were stretched until an initial stable force could be measured (~1mN, with a full-scale deflection of 196 mN). Average tension (=force/mass/length; mN/mm^2^) was calculated and plotted as a function of strain (L/L_o_, where L = the measured length; L_0_ = reference length measured at tissue preparation before tying suture loops at each end). Tissue length (corrected for shortening in culture and for L_0_) was measured at the beginning and end of the experiment and wet tissue mass was measured at the end of the run.

#### Stiffness measurements

Tissue was lengthened stepwise in 0.8 mm increments at 2 minute intervals using an automated micrometer screw driven linear translation stage to which the force transducers were attached. Force transducer output was logged at 5 second intervals. At each step, an initial instantaneous increase in force occurred that then stabilized at a lower level within 2 minutes. Lengthening was reversed when the average force reading exceeded 40 mN (corresponding to ~30mN/mm^2^ tension). At each shortening step, an initial decrease in force occurred that subsequently stabilized. Given these kinetics, tension responses were averaged over the 2 minute lengthening or shortening interval.

Tissue stiffness (mN/mm^2^/mm displacement) was calculated for each bundle from the derivative of the power function tension = A (L/L_0_)^k^, where the scaling factor A is e^b^ with b and k determined by least squares linear regression of double natural log transformed data (r^2^ typically exceeded 0.9 with >5 data pairs). For L/L_o_ = 1, A is the tissue tension (or stress) and Ak is stiffness. In most experiments, tissue was shortened stepwise to baseline, and, after allowing at least 4 minutes for the force output to stabilize, challenged with 1 μM carbachol.

### RNA analysis and immunostaining

RNA was extracted from full thickness mucosa or LSM as previously described^13^. Paraffin embedded full thickness mucosa was used in immunohistochemistry as previously described^13^. Infiltrating intact eosinophils and eosinophil degranulation (i.e., the presence of free cytoplasmic granules and/or tissue deposition of eosinophil granule proteins) were assessed by immunohistochemistry using a mouse monoclonal anti-eosinophil peroxidase antibody (EPX-mAb) as previously described^38,39^. Tissue sections were digitized (Aperio AT Turbo, Leica Biosystems, Buffalo Grove, IL) and images were generated using Aperio ImageScope software (version 11.2.0.780, Aperio Technologies, Vista, CA).

### Eosinophil isolation

Human eosinophils were isolated from peripheral blood by negative selection^40^. Sodium citrate was used as an anticoagulant and hetastarch (6%, StemCell Technologies) was then added to the syringes and red blood cells are allowed to sediment by gravity. The red blood cell depleted layer was then collected and layered over Ficoll-Paque Premium (GE Healthcare), followed by centrifugation. At the end of centrifugation, a granulocyte-enriched population, consisting mainly of neutrophils and eosinophils, was present in a pellet at the bottom of the tube and collected. The collected granulocytes are then washed and incubated with an antibody cocktail with antibodies against CD2, CD14, CD16, CD19, CD56, and glycophorin A (StemCell Technologies). A colloid suspension consisting of magnetic beads that bind to these negative selection antibodies was added, followed by passage over a magnetized column. The effluent contained the purified eosinophil population. Eosinophil purity, assessed by Hema-3 staining (Fisher Scientific) of a cytocentrifuge smear, was routinely greater than 97–99%.

### Mucosal injection of eosinophils

Isolated human eosinophils were kept in IL-5 until use to ensure longevity and injected (80,000 eosinophils per mucosal explant on a transwell chamber) at the serosal surface of mucosa. IL-13 (10 ng/ml) was added to the basal medium and an eotaxin-1 soaked sponge was placed on the epithelium. Mucosal explants were cultured in DMEM:F12 for 24–72 hours, collected, paraffin embedded and stained with hematoxylin and eosin or EPX and analyzed for the location of eosinophils in the mucosa. Sham injection was utilized to assess tissue damage and a time 0 mucosa was collected to assess the starting location of the eosinophils in the tissue.

### Transcription array analysis

The molecular signature was acquired by EoE diagnostic panel (EDP) quantified by a 96 gene RNA expression signature array at Cincinnati Children’s Hospital Medical Center, as reported previously^29^. Briefly, 500 ng of RNA from tissue sample was reverse transcribed to cDNA and subjected to EDP amplification by using the ABI 7900HT qPCR system (Applied Biosystems, Foster City, Calif). To compensate for the long archiving time for some of the samples, a 50% call rate filter was applied to the 96 esophageal genes to focus on informative genes, resulting in a cluster of 60 genes. The data were then imported into GeneSpring (GX 12.6) software for implementation of the analyses.

The transcriptomes of the 10 paired samples from 5 independent donors were compared by using the unsupervised clustering analysis, principal component analysis (PCA), and the paired t test adjusted with the Benjamini-Hochberg method. Clustering was performed by hierarchical clustering design, with the Euclidean distance metric and the Wald’s linkage rules. Condition and gene entity are 2-D clustered in conjunction with expression heat map with red being up-regulation and blue being down-regulation. PCA was used to generate a 3-dimensional plot of the top 3 variance contributors between vehicle and post treatment by fibrotic cocktail. The paired t test adjusted with the Benjamini-Hochberg method was used to compare the treatment effect of paired samples before and after fibrotic cocktail, visualizing by a volcano plot (log2 fold change as x-axis and −log10 adjusted P value as y-axis). A 2-tailed adjusted P-value of less than 0.05 was deemed statistically significant.

### Statistics

GraphPad Prism was used for graphing statistics (unpaired, 2-tailed t-test or Spearman’s correlation coefficient <0.05 was considered significant). Experiments were repeated using >2 donors in triplicates.

## Supplementary information

Supplemental Figures and Tables

## References

[1] Dellon ES (2014). Epidemiology of Eosinophilic Esophagitis. Gastroenterology clinics of North America.

[2] Napier KJ, Scheerer M, Misra S (2014). Esophageal cancer: A Review of epidemiology, pathogenesis, staging workup and treatment modalities. World J Gastrointest Oncol.

[3] Mavi P, Rajavelu P, Rayapudi M, Paul RJ, Mishra A (2012). Esophageal functional impairments in experimental eosinophilic esophagitis. Am J Physiol Gastrointest Liver Physiol.

[4] Hu Y (2014). Increased acid responsiveness in vagal sensory neurons in a guinea pig model of eosinophilic esophagitis. American journal of physiology. Gastrointestinal and liver physiology.

[5] Rieder F (2014). T-helper 2 cytokines, transforming growth factor beta1, and eosinophil products induce fibrogenesis and alter muscle motility in patients with eosinophilic esophagitis. Gastroenterology.

[6] Liacouras Chris A., Furuta Glenn T., Hirano Ikuo, Atkins Dan, Attwood Stephen E., Bonis Peter A., Burks A. Wesley, Chehade Mirna, Collins Margaret H., Dellon Evan S., Dohil Ranjan, Falk Gary W., Gonsalves Nirmala, Gupta Sandeep K., Katzka David A., Lucendo Alfredo J., Markowitz Jonathan E., Noel Richard J., Odze Robert D., Putnam Philip E., Richter Joel E., Romero Yvonne, Ruchelli Eduardo, Sampson Hugh A., Schoepfer Alain, Shaheen Nicholas J., Sicherer Scott H., Spechler Stuart, Spergel Jonathan M., Straumann Alex, Wershil Barry K., Rothenberg Marc E., Aceves Seema S. (2011). Eosinophilic esophagitis: Updated consensus recommendations for children and adults. Journal of Allergy and Clinical Immunology.

[7] Dellon ES (2014). A phenotypic analysis shows that eosinophilic esophagitis is a progressive fibrostenotic disease. Gastrointestinal endoscopy.

[8] Schoepfer AM (2013). Delay in diagnosis of eosinophilic esophagitis increases risk for stricture formation in a time-dependent manner. Gastroenterology.

[9] Nicodeme F (2013). Esophageal distensibility as a measure of disease severity in patients with eosinophilic esophagitis. Clinical gastroenterology and hepatology : the official clinical practice journal of the American Gastroenterological Association.

[10] Aceves SS, Newbury RO, Dohil R, Bastian JF, Broide DH (2007). Esophageal remodeling in pediatric eosinophilic esophagitis. The Journal of allergy and clinical immunology.

[11] Colizzo J. M., Clayton S. B., Richter J. E. (2015). Intrabolus pressure on high-resolution manometry distinguishes fibrostenotic and inflammatory phenotypes of eosinophilic esophagitis. Diseases of the Esophagus.

[12] Menard-Katcher C (2017). Influence of Age and Eosinophilic Esophagitis on Esophageal Distensibility in a Pediatric Cohort. The American journal of gastroenterology.

[13] Rawson R (2016). TGF-beta1-induced PAI-1 contributes to a profibrotic network in patients with eosinophilic esophagitis. The Journal of allergy and clinical immunology.

[14] Nurko S, Rosen R, Furuta GT (2009). Esophageal dysmotility in children with eosinophilic esophagitis: a study using prolonged esophageal manometry. The American journal of gastroenterology.

[15] Roman S (2011). Manometric features of eosinophilic esophagitis in esophageal pressure topography. Neurogastroenterol Motil.

[16] Nennstiel S (2016). High-resolution manometry in patients with eosinophilic esophagitis under topical steroid therapy-a prospective observational study (HIMEOS-study). *Neurogastroenterology and motility : the official journal of the European Gastrointestinal Motility*. Society.

[17] Rossi P (2015). Pp-11 Prolonged Intra-Esophageal Ph Profile and Esophageal Motility in Children with Eosinophilic Esophagitis (Eoe). Journal of pediatric gastroenterology and nutrition.

[18] Korsapati H (2009). Dysfunction of the longitudinal muscles of the oesophagus in eosinophilic oesophagitis. Gut.

[19] Beppu LY (2014). TGF-beta1-induced phospholamban expression alters esophageal smooth muscle cell contraction in patients with eosinophilic esophagitis. The Journal of allergy and clinical immunology.

[20] Muir Amanda B., Dods Kara, Henry Steven J., Benitez Alain J., Lee Dale, Whelan Kelly A., DeMarshall Maureen, Hammer Daniel A., Falk Gary, Wells Rebecca G., Spergel Jonathan, Nakagawa Hiroshi, Wang Mei-Lun (2016). Eosinophilic Esophagitis-Associated Chemical and Mechanical Microenvironment Shapes Esophageal Fibroblast Behavior. Journal of Pediatric Gastroenterology and Nutrition.

[21] Whelan Kelly A, Merves Jamie F, Giroux Veronique, Tanaka Koji, Guo Andy, Chandramouleeswaran Prasanna M, Benitez Alain J, Dods Kara, Que Jianwen, Masterson Joanne C, Fernando Shahan D, Godwin Bridget C, Klein-Szanto Andres J, Chikwava Kudakwashe, Ruchelli Eduardo D, Hamilton Kathryn E, Muir Amanda B, Wang Mei-Lun, Furuta Glenn T, Falk Gary W, Spergel Jonathan M, Nakagawa Hiroshi (2016). Autophagy mediates epithelial cytoprotection in eosinophilic oesophagitis. Gut.

[22] Davis BP (2016). Eosinophilic esophagitis-linked calpain 14 is an IL-13-induced protease that mediates esophageal epithelial barrier impairment. JCI Insight.

[23] Sherrill J D, KC K, Wu D, Djukic Z, Caldwell J M, Stucke E M, Kemme K A, Costello M S, Mingler M K, Blanchard C, Collins M H, Abonia J P, Putnam P E, Dellon E S, Orlando R C, Hogan S P, Rothenberg M E (2013). Desmoglein-1 regulates esophageal epithelial barrier function and immune responses in eosinophilic esophagitis. Mucosal Immunology.

[24] Nguyen N, Fernando S D, Biette K A, Hammer J A, Capocelli K E, Kitzenberg D A, Glover L E, Colgan S P, Furuta G T, Masterson J C (2017). TGF-β1 alters esophageal epithelial barrier function by attenuation of claudin-7 in eosinophilic esophagitis. Mucosal Immunology.

[25] Kalabis J (2012). Isolation and characterization of mouse and human esophageal epithelial cells in 3D organotypic culture. Nature protocols.

[26] Mulvany MJ, Warshaw DM (1979). The active tension-length curve of vascular smooth muscle related to its cellular components. J Gen Physiol.

[27] Ratz PH, Speich JE (2010). Evidence that actomyosin cross bridges contribute to "passive" tension in detrusor smooth muscle. Am J Physiol Renal Physiol.

[28] Doshi Ashmi, Khamishon Rebecca, Rawson Renee, Duong Loan, Dohil Lucas, Myers Stephen J., Bell Braxton, Dohil Ranjan, Newbury Robert O., Barrett Kim E., Kurten Richard C., Aceves Seema S. (2019). Interleukin 9 Alters Epithelial Barrier and E-cadherin in Eosinophilic Esophagitis. Journal of Pediatric Gastroenterology and Nutrition.

[29] Wen Ting, Stucke Emily M., Grotjan Tommie M., Kemme Katherine A., Abonia J. Pablo, Putnam Philip E., Franciosi James P., Garza Jose M., Kaul Ajay, King Eileen C., Collins Margaret H., Kushner Jonathan P., Rothenberg Marc E. (2013). Molecular Diagnosis of Eosinophilic Esophagitis by Gene Expression Profiling. Gastroenterology.

[30] Ohnmacht C, Pullner A, van Rooijen N, Voehringer D (2007). Analysis of eosinophil turnover *in vivo* reveals their active recruitment to and prolonged survival in the peritoneal cavity. Journal of immunology.

[31] Wen T, Besse JA, Mingler MK, Fulkerson PC, Rothenberg ME (2013). Eosinophil adoptive transfer system to directly evaluate pulmonary eosinophil trafficking *in vivo*. Proceedings of the National Academy of Sciences of the United States of America.

[32] Kwiatek MA (2011). Mechanical properties of the esophagus in eosinophilic esophagitis. Gastroenterology.

[33] Park SY, Youm JH, Jung KC, Sohn UD (2008). Inhibitory effect of hypochlorous acid on lower esophageal sphincter tone relaxation by vasoactive intestinal peptide. Archives of pharmacal research.

[34] Liu JF, Lu HL, Wen SW, Wu RF (2011). Effects of acetylcholine on sling and clasp fibers of the human lower esophageal sphincter. J Gastroenterol Hepatol.

[35] Tottrup A, Forman A, Uldbjerg N, Funch-Jensen P, Andersson KE (1990). Mechanical properties of isolated human esophageal smooth muscle. The American journal of physiology.

[36] Kwiatek MA, Pandolfino JE, Hirano I, Kahrilas PJ (2010). Esophagogastric junction distensibility assessed with an endoscopic functional luminal imaging probe (EndoFLIP). Gastrointestinal endoscopy.

[37] Southard JH, Belzer FO (1995). Organ preservation. Annu Rev Med.

[38] Protheroe C (2009). A novel histologic scoring system to evaluate mucosal biopsies from patients with eosinophilic esophagitis. Clinical gastroenterology and hepatology : the official clinical practice journal of the American Gastroenterological Association.

[39] Wright, B. L. *et al*. Normalized serum eosinophil peroxidase levels are inversely correlated with esophageal eosinophilia in eosinophilic esophagitis. *Diseases of the esophagus : official journal of the International Society for**Diseases of the Esophagus/I.S.D.E* **31**, 10.1093/dote/dox139 (2018). 10.1093/dote/dox139 PMC7373170 29228243

[40] Akuthota P, Capron K, Weller PF (2014). Eosinophil purification from peripheral blood. Methods Mol Biol.

