# Supplementary material for: Development and Application of a Functional Human Esophageal Mucosa Explant Platform to Eosinophilic Esophagitis
Source: Sci Rep. 2019 Apr 17;9:6206. doi: 10.1038/s41598-019-41147-8 (PMC6470157; doi:10.1038/s41598-019-41147-8)
Supplement: Supplementary file 1 — Supplemental Figures and Tables [file 41598_2019_41147_MOESM1_ESM.pdf]

Supplemental Figures and Tables

Development and Application of a Functional Human Esophageal Mucosa Explant Platform to Eosinophilic Esophagitis

Richard C. Kurten, PhD*[1,2,3], Renee Rawson, BS[4], Tetsuo Shoda, MD, PhD[5], Loan D. Duong BS[4], Dolapo Adejumobi, BS[1,2,3], Rebecca Levy, MD[3,6], Robert O. Newbury, MD[6,7], Marc Rothenberg, MD, PhD[5], Praveen Akuthota[8,11], MD, Benjamin L. Wright, MD[9], Ranjan Dohil MD[4,10], Stacie M. Jones, MD[2,3], Seema S. Aceves, MD, PhD*[2,4,11]

[1]Department of Physiology & Biophysics, [2]Division of Allergy & Immunology, [3]Arkansas Children's Research Institute and University of Arkansas for Medical Sciences, Little Rock, Arkansas, [4]Department of Pediatrics, University of California, San Diego, [5]Department of Pediatrics, Division of Allergy, Immunology, Cincinnati Children's Hospital Medical Center, University of Cincinnati, [6]Department of Pathology, [7]Department of Pediatrics, University of California, San Diego and Rady Children's Hospital, San Diego, [8]Division of Pulmonary, Critical Care, and Sleep Medicine, [9]Division of Allergy, Asthma and Clinical Immunology, Department of Medicine, Mayo Clinic Arizona, Scottsdale, Arizona, Division of Pulmonology, Phoenix Children's Hospital, Phoenix, Arizona, [10]Division of Gastroenterology, [11]Department of Medicine, University of California, San Diego

Funding Sources: NIH/NIAID AI092135 (S.S.A, R.C.K.), NIH/NIAID AI135034 (S.S.A.) NIH/NCRR/NCATS UL1TR000039 (R.C.K.), Claudia and Steve Strange Family Fund (S.M.J.), NIH U19 AI070235 (MR, SSA), NIH R01 AI124355 (MR), R37 A1045898 (MR), the Campaign Urging Research for Eosinophilic Disease (CURED) Foundation (MR), the Sunshine Charitable Foundation and its supporters (MR), Denise A. Bunning and David G. Bunning (MR).

Supplemental Figure 1

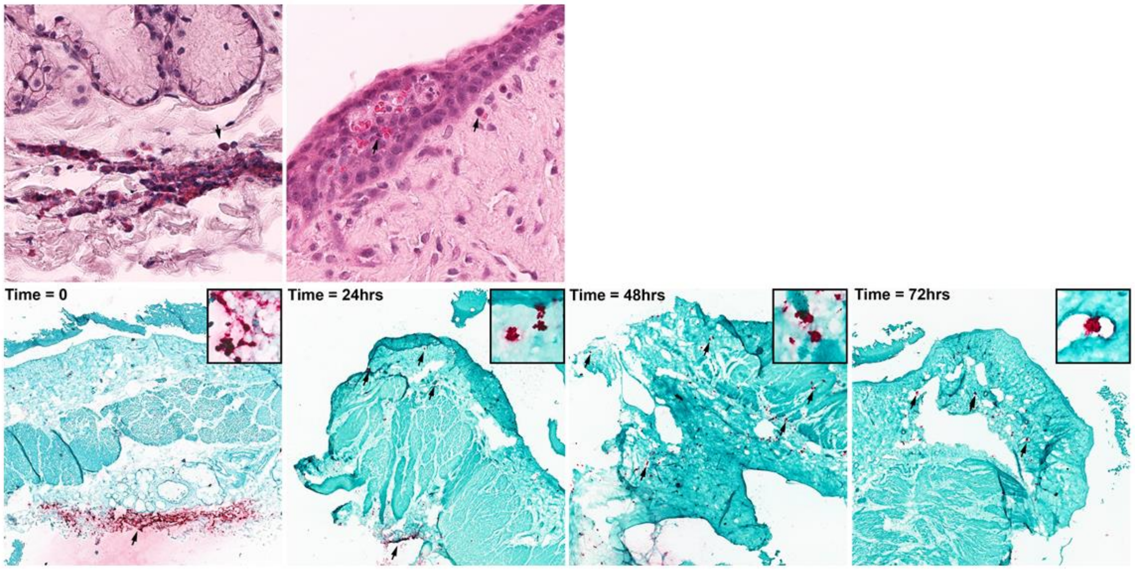

Figure S1. Infiltration of eosinophils into ex vivo human mucosa. High power images of H&E stained mucosa at 0 hours show initial injection site (arrows, top left panel) and infiltration of eosinophils into the lamina propria and epithelial space at 24 hours (arrows, top right panel). Eosinophil peroxidase (EPX) staining to confirm cells as eosinophils (red) at time =0 demonstrates the injection site (bottom left panel, arrow). At 24, 48 and 72 hours post injection (arrowheads) eosinophils are present in the mucosa (bottom right panels). Insets show high power images of EPX positive eosinophils. See methods in main manuscript for experimental details.

Supplemental Figure 2

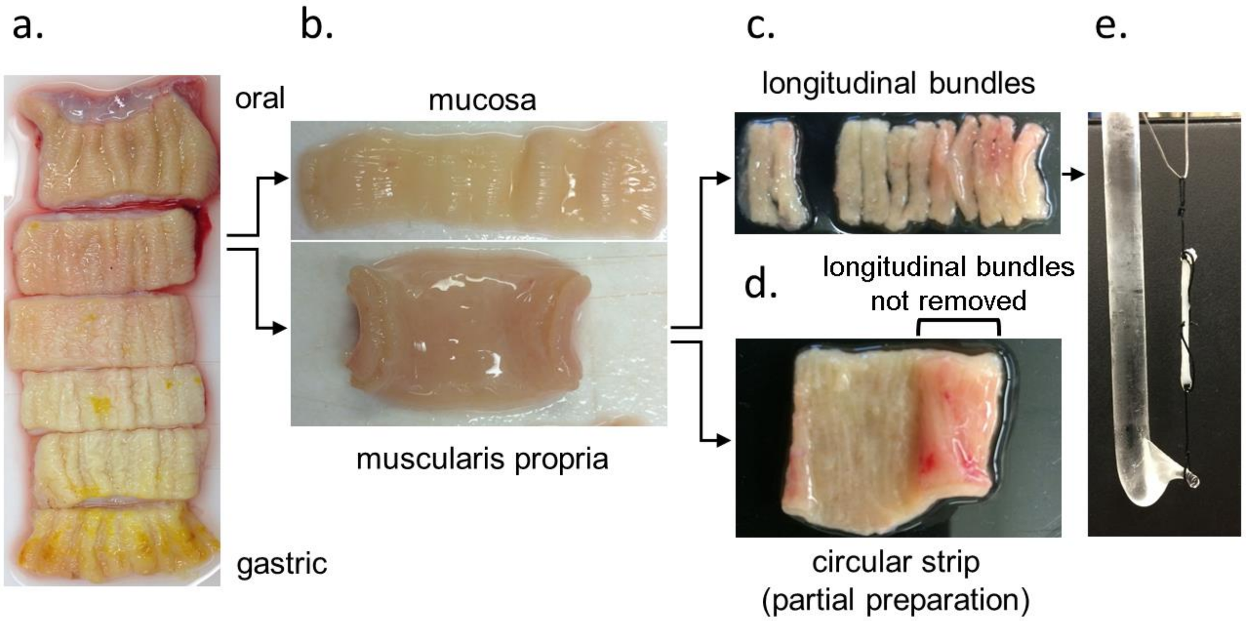

Figure S2. Preparation of Esophageal tissues. a. Distal half of esophagus opened longitudinally, laid mucosal side up and cut into ~15mm long strips. b Separation of mucosa and muscularis propia from a single strip. The mucosa strip was further cut into ~16 1-2mm wide strips with a scalpel c. Sharp dissection of longitudinal smooth muscle bundles from the muscularis propia. d. Partially competed circular smooth muscle preparation from which 15mm long strips were prepared. e. Longitudinal smooth muscle bundle secured with silk sutures at each end to a fixed glass post at the bottom and to a wire at the top leading to movable force transducer. The preparation is lowered into a temperature-controlled bath for assay.

Supplemental Table 1

Donor Demographics

AGE	Mean 33.1 StdDev 14.9 n=25	
SEX	Male	52%
	Female	48%
RACE	Caucasian	52%
	African American	20%
	Hispanic	12%
	unknown or other	16%

Supplemental Table 2. Donor Characteristics and Use in Experiments

*Used as the positive control for Figure 5 and not utilized in any other experiment

	Donor Number	Age	Race	Sex	COD	Eos per hpf	BZH (0-2)	FS (0-3)	DIS (0/1)	Figure
1	1	37	U	F	CVA/Stroke	-	-	-	-	Figure 1a
2	2	52	AA	F	CVA/Stroke	-	-	-	-	Figure 1a
	2	52	AA	F	CVA/Stroke	-	-	-	-	Figure 1b
3	3	53	C	M	CVA/Stroke	-	-	-	-	Figure 1a
4	4	16	AA	M	CVA/Stroke	-	-	-	-	Figure 1c
5	9	60	C	F	CVA/Stroke	-	-	-	-	Figure 1d
	9	60	C	F	CVA/Stroke	-	-	-	-	Figure 1f
6	25	13	C	F	HT-SIGSW	-	-	-	-	Figure 4a
7	30	42	H	F	CVA/Stroke	-	-	-	-	Figure 1e
8	31	18	C	F	Asthma	-	-	-	-	Figure 3a
	31	18	C	F	Asthma	-	-	-	-	Figure 3b
9	32	9	AA	F	CVA	-	-	-	-	Figure 3a
	32	9	AA	F	CVA	-	-	-	-	Figure 3b
10	33	41	AI	M	HT - Blunt Injury - MVA	-	-	-	-	Figure 3a
	33	41	AI	M	HT - Blunt Injury - MVA	-	-	-	-	Figure 3b
11	34	20	C	F	HT - Blunt Injury - MVA	-	-	-	-	Figure 3a
	34	20	C	F	HT - Blunt Injury - MVA	-	-	-	-	Figure 3b
12	35	53	C	M	CVA	-	-	-	-	Figure 3a
	35	53	C	M	CVA	-	-	-	-	Figure 3b
13	36	16	AA	M	Anoxia-Asphyxiation	0	2	1	3	Figure 3a
	36	16	AA	M	Anoxia	-	-	-	-	Figure 3b
14	37	25	C	M	HT - Blunt Injury - MVA	0	0	0	0	Figure 3a
	37	25	C	M	HT - Blunt Injury - MVA	-	-	-	-	Figure 3b
15	40	28	C	M	Anoxia-Drug Intoxication	4	1	2?	5	Figure 4c
16	43	34	C	M	Blunt Injury - MVA	-	-	-	-	Figure 3a
	43	34	C	M	Blunt Injury - MVA	-	-	-	-	Figure 3b
17	50	14	H	F	GSW to the Head	-	-	-	-	Figure 2g-h
18	55	36	AA	F	CVA/Stroke	0	0	0	0	Figure 1g
	55	36	AA	F	CVA/Stroke	-	-	-	-	Figure 2a-f
19	56	39	C	M	unknown	0	0	0	0	Figure 6
20	57	49	C	F	CVA/Stroke	0	0	0	1	Figure 5
21	61	36	U	M	unknown	0	0	0	0	Figure 5
22	63	45	C	F	Anoxia/ Drug Intoxication	0	0	0	0	Figure 5
23*	77	30	C	M	Anoxia/CVA	45	2	0	0	Figure 5
24	87	16	H	M	HT-SIGSW	0	0	0	0	Figure 5
25	102	45	C	M	CVA/ICH	0	0	0	0	Figure 5

Abbreviations: A – African American; C – Caucasian; CVA – cerebrovascular accident; F- Female; GSW –gunshot wound H- Hispanic; HT – head trauma; ICH - intracerebral hemorrhage; M- Male; MVA – motor vehicle accident; SIGSW – self inflicted gunshot wound; U – unknown; BZH - basal zone hyperplasia; LP FS – lamina propria fibrosis score; DIS- dilated intercellular spaces
